# A Portable Biosensor for 2,4-Dinitrotoluene Vapors

**DOI:** 10.3390/s18124247

**Published:** 2018-12-03

**Authors:** Marc Prante, Christian Ude, Miriam Große, Lukas Raddatz, Ulrich Krings, Gernot John, Shimshon Belkin, Thomas Scheper

**Affiliations:** 1 Institute of Technical Chemistry, Leibniz University of Hannover, 30167 Hannover, Germany; prante@iftc.uni-hannover.de (M.P.); raddatz@iftc.uni-hannover.de (L.R.); scheper@iftc.uni-hannover.de (T.S.); 2 Institute of Food Chemistry, Leibniz University of Hannover, 30167 Hannover, Germany; miriam.grosse@lci.uni-hannover.de (M.G.); krings@lci.uni-hannover.de (U.K.); 3 PreSens Precision Sensing GmbH, 93053 Regensburg, Germany; g.john@presens.de; 4 Department of Plant and Environmental Sciences, The Alexander Silberman Institute of Life Sciences, The Hebrew University of Jerusalem, 9190401 Jerusalem, Israel; shimshon.belkin@mail.huji.ac.il

**Keywords:** biosensor, landmine detection, chemical vapor signature, biological sensor, bioluminescence, explosive material

## Abstract

Buried explosive material, e.g., landmines, represent a severe issue for human safety all over the world. Most explosives consist of environmentally hazardous chemicals like 2,4,6-trinitrotoluene (TNT), carcinogenic 2,4-dinitrotoluene (2,4-DNT) and related compounds. Vapors leaking from buried landmines offer a detection marker for landmines, presenting an option to detect landmines without relying on metal detection. 2,4-Dinitrotoluene (DNT), an impurity and byproduct of common TNT synthesis, is a feasible detection marker since it is extremely volatile. We report on the construction of a wireless, handy and cost effective 2,4-dinitrotoluene biosensor combining recombinant bioluminescent bacterial cells and a compact, portable optical detection device. This biosensor could serve as a potential alternative to the current detection technique. The influence of temperature, oxygen and different immobilization procedures on bioluminescence were tested. Oxygen penetration depth in agarose gels was investigated, and showed that aeration with molecular oxygen is necessary to maintain bioluminescence activity at higher cell densities. Bioluminescence was low even at high cell densities and 2,4-DNT concentrations, hence optimization of different prototypes was carried out regarding radiation surface of the gels used for immobilization. These findings were applied to sensor construction, and 50 ppb gaseous 2,4-DNT was successfully detected.

## 1. Introduction

Many explosive remnants of current and past armed conflicts can be still found today scattered all over the world. It is estimated that there are over 110 million landmines in 70 different countries [1]. Anti-personnel landmines are cheap and no expertise is required for their simple deployment [1,2]. Safe and robust detection methods are necessary to find and remove the explosives. The most common method is the use of a metal detector which is basically unchanged since its invention in 1943. However, a lot of modern mines are made of plastic revealing the major drawback of this detection strategy, since it indirectly detects not the explosive itself but only the mine’s housing. Detection using a metal detector is thus limited and should be replaced with a safer and modern approach which targets the explosive chemical itself [3].

In the past years innovative approaches regarding landmine detection methods were examined which aim at the detection of the chemical compounds within the landmine, rather than the landmine housing. The principle of these sensors is based on the detection of volatile chemicals such as 2,4-DNT leaking from the landmines, by electromechanical, piezoelectric or spectroscopic techniques [4,5,6]. Besides these, biological detectors are in the current research interest. Dogs or giant-pouched rats, for example, can be trained to detect the volatile chemicals from buried landmines [7].

Another option for mine detection is based on the use of biosensors: hardware devices harboring a biological entity which selectively reacts with the target compounds [8]. The ensuing reaction is detected by a transducer (optical-, piezoelectric-, electrochemical- and thermal-detection transduction are the most common) and converted and amplified to a quantifiable electrical signal which is proportional to the concentration of the chemical [9,10]. Over the past few years, biosensors in which the biological entity is a live cell (whole-cell biosensors) have received considerable attention, in view of the fact that the enzymes that catalyze the specific reaction are already in their ideal environment and more stable [11,12]. Combining this with the existing approaches of detecting leaking vapors from landmines a new biosensor for the landmine detection is feasible, with potential low cost production and eliminating the risks involved to people or animals.

In previous studies carried out by Yagur-Kroll et al. a bioluminescent bioreporter system for the volatile compound 2,4-DNT using recombinant *Escherichia coli* cells was established. The bioreporter reacted to 2,4-DNT, triggering the expression of *luxCDABE* genes, thus generating a dose-dependent luminescent signal [13,14]. The bacterial luciferase, encoded by the *luxAB* genes, oxidizes a long-chain aldehyde and a reduced flavin mononucleotide and emits light at 490 nm in an oxygen-dependent reaction Equation (1):

FMNH_2_ + RCHO + O_2_ → FMN + RCOOH + H_2_O + *hv* (490 nm) (1)

**Figure 1:**
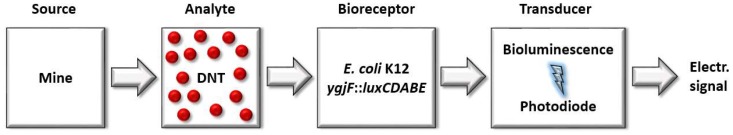
Scheme of the proposed biosensor for the detection of 2,4-DNT evaporating from buried landmines. The production of luciferase in recombinant *E. coli* cells is triggered by 2,4-DNT, which leads to a bioluminescence emission peak at 490 nm. A silicone photodiode acts as a transducer and converts the bioluminescence into a quantifiable, electrical signal.

This bioreporter strain is the biological element which enables the construction of a photodiode-based optical biosensor described in this communication (Figure 1). Cell immobilization is a crucial step in the development of whole-cell biosensors. However, information on the behavior of immobilized recombinant bioluminescent is scarce [12,15,16]. In the present article we report the construction of a first optical 2,4-DNT biosensor prototype employing immobilized recombinant cells for the detection of buried landmines.

At first, the general properties of the bacterial bioreporter regarding different parameters were examined. It is crucial to investigate the effects of external factors like temperature and oxygen on the bioluminescence regarding the later sensor development. Moreover, the effect of immobilization on luciferase activity has received little attention in literature, which was another part of this study. As soon as the general properties of the luciferase and the behavior of various influencing factors were clarified, the actual sensor design and evaluation followed. This final step included sensor measurements with 2,4-DNT both in the liquid and gas phase.

## 2. Materials and Methods

### 2.1. Chemicals

2,4-Dinitrotoluene (99% *w*/*w*) was purchased from Sigma-Aldrich Corporation (St Louis, MO, USA) and dissolved in 80% acetonitrile (*v*/*v*) yielding a final 2,4-DNT concentration of 0.1 g∙L^−1^, unless otherwise stated.

### 2.2. Growth Conditions

*Escherichia coli* K12 MG1655 cells containing a fusion between the *luxCDABE* operon and the *yqjF* gene promoter, previously reported by Yagur-Kroll et. al [13], were used as a reporter strain. The cells were cultivated in modified 100 mL shake flasks containing modified lysogeny broth (LBB; 10 g∙L^−1^ Tryptone, 5 g∙L^−1^ yeast extract, 1 g∙L^−1^ NaCl, 5 g∙L^−1^ K_2_HPO_4_, 3 g∙L^−1^ KH_2_PO_4_, pH 7.6) with shaking (200× rpm) at 37 °C unless otherwise stated. Carbenicillin was used at a final concentration of 100 µg∙mL^−1^ for selection of cells containing the plasmid. Induction of luciferase production was accomplished by adding 2,4-DNT (in 80% acetonitrile (*v*/*v*)) at a final concentration of 100 mg∙L^−1^ to the shaking flask during exponential growth.

### 2.3. Cell Immobilization

The cells were harvested after 3 h of induction and centrifuged at 10,000× *g* at 20 °C for 10 min. The resulting pellet was suspended in LBB medium (50 mL) to obtain a final optical density at 600 nm (OD_600_) = 10 unless otherwise stated (8.0 × 10^9^ cells per mL). Agarose (1% (*w*/*v*), 40 °C) was added and 900 µL of the solution was poured into a casting mold resulting in a 1% (*w*/*v*) agarose hydrogel with a final cell count of 3.6 × 10^9^ cells per mL. For cell immobilization in alginate, alginate was dissolved in deionized water (2% *w*/*v*, final concentration) and the solution was mixed with an equal volume of fresh LBB medium containing cells at OD_600_ = 10, resulting in a final cell count of 4.0 × 10^9^ cells per mL. The mixture was dripped into 1% (*w*/*w*) solution of calcium chloride and stirred for 25 min. The resulting alginate beads were used to measure bioluminescence in prototype 1.0, agarose disks were used for measurements in prototype 2.0.

### 2.4. Design and Construction of Biosensors

The biosensor prototypes were designed using CAD software Autodesk Inventor 2015 (San Rafael, CA, USA) and 3D-printed with a multi jet ProJet MJP 2500 Plus (3D-Systems, Rock Hill, SC, USA) and VisiJet M2-RCL epoxy resin (3D-Systems, Rock Hill, SC, USA) as the printing material. Parts were post-processed by incubations in heat steam (60 °C, 30 min) and an oil bath (50 °C, 30 min), and rinsed with W5 detergent solution (Lidl, Neckarsulm, Germany).

### 2.5. Oxygen Dependency

**Figure A1:**
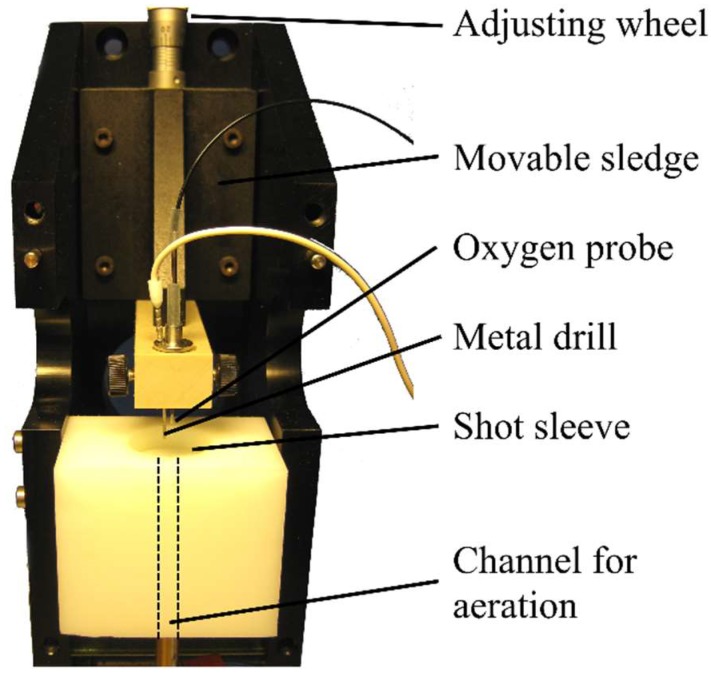
Micromanipulator device to measure the oxygen saturation in different hydrogel-depths. The oxygen probe was fixated in a movable sledge which was moved in defined 50 µm distances by an adjusting wheel. The hydrogel containing the bacterial cells was casted in a shot sleeve, under which a channel for aeration with oxygen was located. After the gel was casted, molecular oxygen was introduced through the channel and the resulting oxygen penetration depth was recorded with the oxygen probe OXY1-ST-pST7.

**Figure A2:**
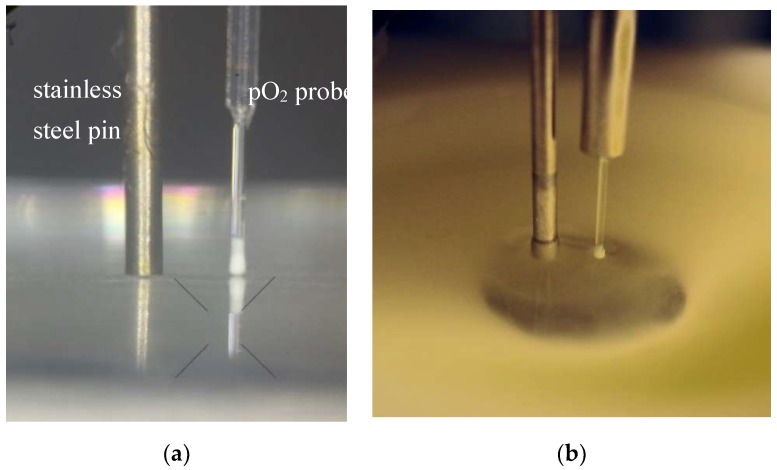
Exemplary optical zeroing of the oxygen probe on a plane glass surface. A microscope was used to verify the position of the oxygen probe and the aluminum bolt which was used to zero the micromanipulator device shown (**a**). Picture of the oxygen probe which was incorporated into the hydrogel to measure oxygen saturation in different gel depths (**b**).

Oxygen saturation in agarose hydrogels was measured using an OXY1-ST oxygen meter and IMP-PSt7 oxygen microsensor (PreSens Precision Sensing GmbH, Regensburg, Germany). The probe was fixated in a micromanipulator device which enables the probe to be positioned at defined heights up to an accuracy of 50 µm. An aluminum bolt and a drill were connected to a multimeter to determine the zero point of the oxygen probe (Figure A1). When the drill got in contact with the bottom part of the shot sleeve a change in the resistor could be detected. The adjustment was verified using a stereo-microscope (Figure A2). Afterwards, the aluminum bolt was removed and an oxygen permeable membrane was placed on top of the emerging hole. Several distances between the lower end of the hydrogel and the tip of the oxygen probe were adjusted and hydrogels were casted. The hydrogels were overlaid with liquid paraffin to ensure that oxygen reaches the cells only through the aeration channel. The experiments were repeated three times and the resulting error bars were created using the standard deviation between measurements.

### 2.6. Determination of Bioluminescence by a Fluorescence Spectrophotometer

Bioluminescence was determined in a F7000 Fluorescence spectrophotometer (Hitachi Ltd. Corporation, Tokyo, Japan) at λ = 490 nm. The excitation was turned off and the excitation slit was closed. Integration time of the device was set to 0.2 s with a delay of 0.5 s. The photomultiplier (PMT) voltage was set to 700 V.

### 2.7. On-line Measurement of Oxygen, Temperature and Bioluminescence

**Figure A3:**
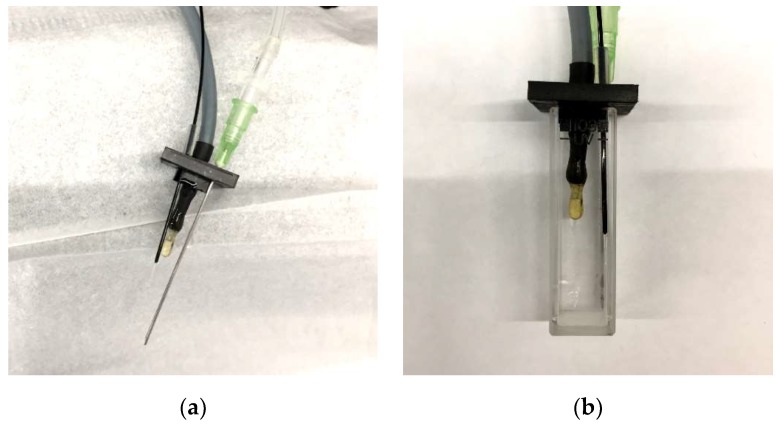
Modifications of the UV-cuvette lid to monitor oxygen and temperature and introduce molecular oxygen simultaneously (**a**). Exemplary picture of the lid mounted on a UV-cuvette ready for measurements (**b**).

A standard fused-silica UV-cuvette was equipped with a temperature probe (Pt-100) and the implantable oxygen micro sensor IMP-PSt7 was connected to an OXY-1 ST (Figure A3). The oxygen saturation was measured in % absolute saturation (% a.s.). The UV-cuvette was placed next to a heatable element to enable a constant temperature regulation of the liquid in the cuvette. A syringe was connected to introduce oxygen into the liquid. Furthermore, a Pt-100 was added to measure the temperature of the liquid. The cuvette was placed in a fluorescence spectrometer (Hitachi F-7000, Hitachi Ltd. Corporation, Tokyo, Japan) to measure bioluminescence at 490 nm. A special cuvette lid was constructed, incorporating three drilled holes for different probes. The cuvette including a probe for temperature, oxygen saturation and a syringe used for aeration was placed in the spectrometer. Molecular oxygen was continuously introduced into the cuvette at a flow rate of 5 mL∙min^−1^ and the resulting effect on bioluminescence was measured. Simultaneously, the measurement of oxygen saturation and temperature was started. The same procedure was repeated using nitrogen instead of oxygen.

### 2.8. Measurement Software for the 2,4-DNT Biosensor

The photodiode SD112-45-11-221-ND (Luna Optoelectronics, Roanoke, VA, USA) used in the sensor prototypes was connected to a transmitter unit located inside an aluminum case and connected to a computer. The measuring software was developed in-house using ProfiLab Expert 5 (Meilhaus Electronics GmbH, Alling, Germany).

### 2.9. GC-MS Measurements of Gaseous 2,4-DNT

**Figure A4:**
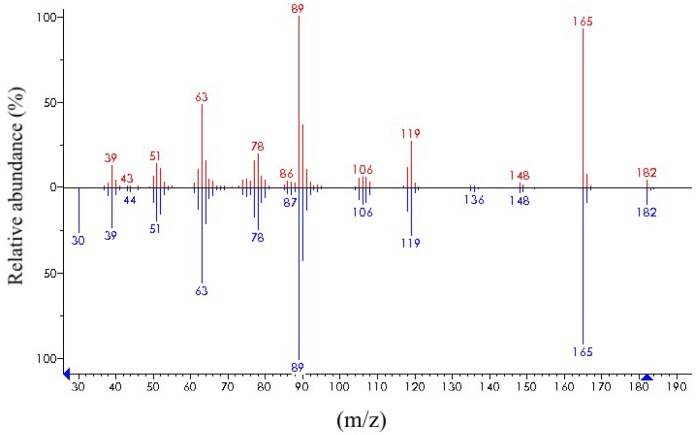
Mass to charge spectrum of 2,4-dinitrotoluene at a retention time of 24.7 min detected with TDS silicone twisters and GC-MS.

To detect volatile 2,4-DNT modified EG/silicone stir bars (Twister, Gerstel GmbH & Co. KG, Muelheim an der Ruhr, Germany) were used to extract the nonpolar molecule. The glass tube with the loaded twister was transferred into a thermal desorption system (TDS, Gerstel GmbH & Co. KG) of a TDS-CIS-GC-MS (Agilent Technologies, Santa Clara, CA, USA). The 5975B Agilent GC was equipped with a cold injection system (CIS, Gerstel GmbH & Co. KG), a 30 m × 0.32 VF-5ms capillary column (Agilent J&W GC columns, Santa Clara, CA, USA) and connected to an Agilent 6890N mass selective detector (MSD). Operation conditions were as follows: the TDS was heated at a rate of 60 °C min^−1^ from 20 to 250 °C with a He desorption flow rate of 40 mL min^−1^ and held for 3 min. During the desorption period the CIS was operated in the split mode at −10 °C, thereby cryofocusing thermally desorbed analytes. Gas chromatography – mass spectrometry(GC-MS) analysis started with ballistic heating (12 °C s^−1^) of the CIS in the splitless mode with a He column flow rate of 1.3 mL min^−1^ and oven heating from 40 °C to 325 °C at 8 °C min^−1^. The mass range of the MSD was set from m/z 33 to 300 and the EI-source operated at 70 eV and 200 °C (see Figure A4 for mass to charge spectrum).

### 2.10. CalibrationTable

**Figure A5:**
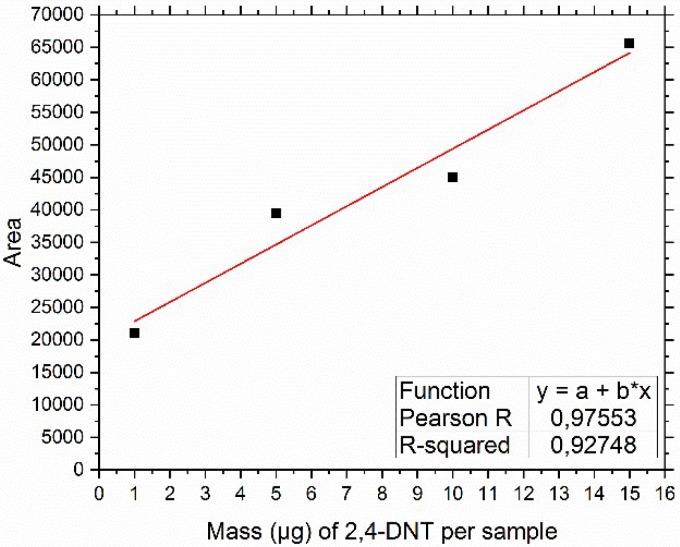
Calibration row for the detection of gaseous 2,4-DNT using GC-MS as stated in Material and Methods 2.10.

A four point calibration series using different concentrations of aqueous 2,4-DNT was created and measured using a GC-MS at the mentioned operation conditions. A linear regression with a regression coefficient of 0.95 was obtained (see Figure A5). The resulting regression Equation is shown in Equation (2). The area determined by GC-MS is shown as x, y is the mass of 2,4-DNT per sample in µg:

(2) y= 2948.3 x + 19920

### 2.11. Determination of the 2,4-DNT Equilibrium Concentration

**Figure A6:**
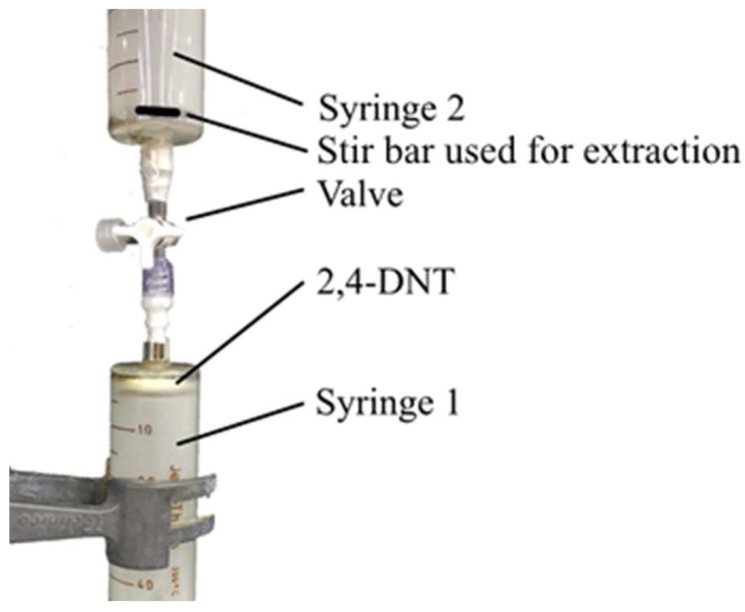
Experimental set-up to create a 2,4-DNT saturated headspace in a defined volume. Two glass syringes were connected using a closed valve. 2,4-DNT (150 mg) was solved in deionized water (2 mL), transferred in syringe 1 and incubated for 24 h. On the next day, the valve was opened and the whole headroom (50 mL) was transferred into Syringe 2 containing a TDS twister. The twister was then analyzed using GC-MS.

Deionized water (2 mL) was added to 2,4-DNT (150 mg) and transferred into a glass-syringe (syringe 1) with a total volume of 50 mL. Syringe 1 was then connected to another syringe (syringe 2) using a closed valve. An adsorptive stir bar was placed in syringe 2 to extract gaseous 2,4-DNT. This setup was incubated at 25 °C for 24 h to guarantee a 2,4-DNT saturated atmosphere in syringe 1. Afterwards, the gas phase of syringe 1 containing the 2,4-DNT saturated air was transferred into syringe 2 containing the stir bar and incubated at 25 °C for 24 additional hours to extract 2,4-DNT (see Figure A6 for experimental set-up). The 2,4-DNT adsorbed to the stir bar was then analyzed using GC-MS as stated previously.

## 3. Results

### 3.1. Influence of Oxygen on Bioluminescence

**Figure 2:**
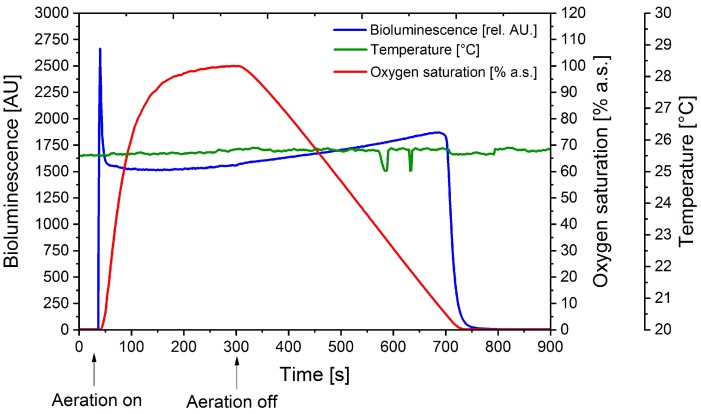
Determination of the influence of oxygen concentration on bioluminescence emitted by the cells in a fused-silica cuvette. A cell culture of the reporter strain carrying the *yqjF*::*luxCDABE* fusion, pre-induced by 100mg∙L^−1^ 2,4-DNT, was standardized to an OD_600_ = 5. Temperature was kept constant at 25 °C. Over the course of the experiment oxygen saturation, temperature and bioluminescence were measured and the cuvette was aerated with molecular oxygen where marked. Bioluminescence data is provided in relative arbitrary units (AU).

A quartz cuvette was equipped with a PT-100 temperature sensor and oxygen probe, as well as with a syringe for aeration. Liquid culture of the bacterial bioreporter (OD_600_ = 5) was transferred into the cuvette, aeration with molecular oxygen was started (5 mL∙min^−1^) and bioluminescence was measured continuously using a fluorescence spectrophotometer (Figure 2). Oxygen aeration led to an initial bioluminescence peak with an intensity of 2650 AU before the oxygen probe detected a change in the oxygen saturation.

After 30 s the signal returned to a steady baseline at about 1650 rel. AU while aeration is continued. After turning off the aeration the signal was relatively stable until a critical oxygen saturation of about 4% a.s. was reached. At this point the oxygen concentration was apparently too low for the luciferase to maintain the bioluminescence reaction.

To preclude that the increase of bioluminescence is simply caused by cell movement and consequently quorum sensing mechanisms the same experiment was repeated using nitrogen gassing instead of molecular oxygen. When repeating the same experiment with nitrogen no significant bioluminescence signal could be detected. As expected, bioluminescence strongly depends on the oxygen saturation. It is also evident, that very low oxygen concentrations (≥4% a.s.) are sufficient to maintain optimal bioluminescence emission.

### 3.2. Influence of Temperature on Bioluminescence

**Figure 3:**
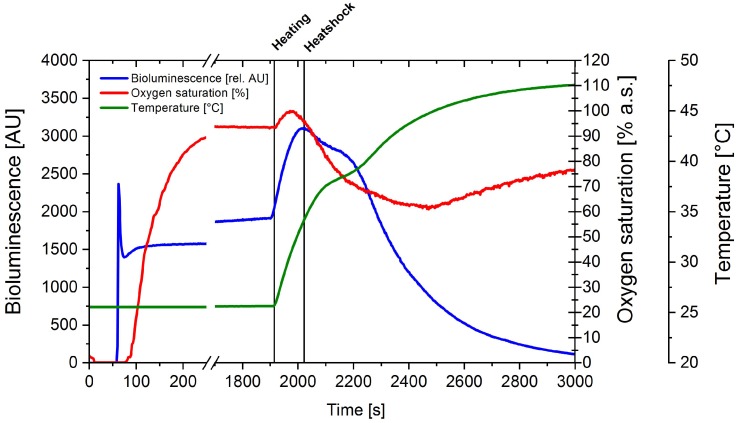
Determination of the influence of temperature on bioluminescence emitted by the cells in a fused-silica cuvette. A cell culture of the reporter strain carrying the *yqjF*::*luxCDABE* fusion, pre-induced by 100mg∙L^−1^ 2,4-DNT, was standardized to an OD_600_ = 5. Over the course of the experiment oxygen saturation, temperature and bioluminescence were measured and the cuvette was aerated with molecular oxygen until the saturation reached a constant signal. The cuvette was then heated to 47 °C.

The same experimental design as in the previous subsection was set up for the determination of the influence of the temperature in bioluminescence. A temperature gradient was applied to the cell suspension and temperature, oxygen saturation and bioluminescence were simultaneously measured (Figure 3). Followed by an initial bioluminescence peak, a relatively constant signal of 1500 AU was observed, which slowly increased to 1800 AU over the course of 2000 s. Over this period the temperature was kept constant at 26 °C and oxygen saturation at 100% a.s.

After the oxygen saturation reached a steady signal at 1800 s a temperature gradient was applied to heat up the liquid to 47 °C. With an increasing temperature bioluminescence also increased up to a maximum of 3000 AU at 34 °C. At higher temperatures the bioluminescence rapidly decreased. A decrease in oxygen saturation was also observable after starting the heating process at 2000 s, as solubility of oxygen in liquid decreased at higher temperatures. Moreover, the oxygen uptake rate increases with an increasing temperature, which results in a higher metabolic activity and oxygen consumption. However, after 2500 s the oxygen saturation did not decline further but increased. This effect could be observed at a temperature of 44 °C where most likely respiration declined due to the sub-optimal temperature for the cells.

### 3.3. Oxygen Distribution in Hydrogels

**Figure 4:**
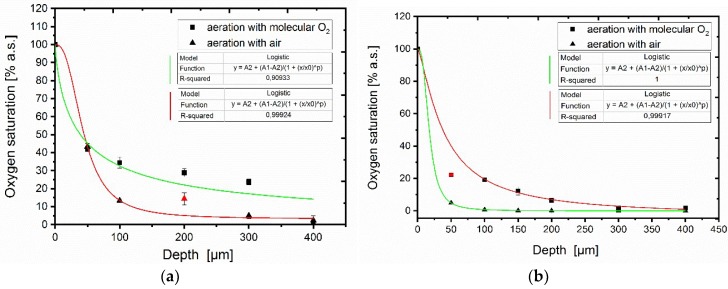
Oxygen depth profile in agarose (1% (*w*/*v*)) encapsulating the bioluminescent strain containing the *yqjF*::*luxCDABE* fusion at two cell densities. An oxygen microprobe was polymerized into the gel and the bottom side of the gel was aerated either with oxygen or air and the oxygen saturation was measured once it reached a stationary point. The distance between the lower edge of the shot sleeve (see Figure A7) and the tip of the oxygen probe was used to display oxygen penetration OD_600_ = 1 (**a**), OD_600_ = 5 (**b**).

Oxygen supply in hydrogels is important for cell vitality as well as for the luciferase activity, and consequently for the sensor signal. To determine optimal gel thickness for cell immobilization, an oxygen diffusion profile in a hydrogel containing the cells was measured. Using an oxygen probe the hydrogel was either aerated with air or with molecular oxygen through the aeration channel (Figure A1 and Figure A9). Different cell concentrations (OD_600_ = 1 and 5) were used to analyze the resulting pO_2_ profile once it reached a stable signal (Figure 4). An agarose gel (1% (*w*/*v*)) containing cells with a final OD_600_ = 1 was cast and the resulting oxygen saturation at different gel depths was measured (Figure 4a).

At the outer layers of the hydrogel (50 µm) aeration with air is sufficient to exceed the critical oxygen saturation threshold of 4% a.s. necessary (Figure 2) to maintain a stable bioluminescence signal. At increasing depths oxygen concentration quickly diminished and was observed to be 0% a.s. at 400 µm. When aeration with molecular oxygen was initiated the oxygen profile shifted upwards and oxygen saturation up to 2% a.s. was measured at depths up to 400 µm. The same experiment was repeated using an agarose gel containing a final OD_600_ = 5 (Figure 4b).

At a depth of 50 µm only 5% a.s. could be observed. Aeration of the hydrogel with molecular oxygen maintains the critical oxygen saturation threshold of ≥4% a.s. up to depths of 200 µm. Aeration with air is not sufficient for cell densities OD_600_ ≥ 5 and should be considered for following experiments. The determined oxygen threshold for maintaining a stable bioluminescence signal was reached in the optical density OD_600_ = 5 agarose disk only at a depth of 300 µm with molecular oxygen aeration. Lower cell densities (e.g., OD_600_ = 1) facilitate the possibility to cast gels with thicknesses up to 800 µm which are better to handle in the final application. However, a lower cell density results in a lower sensor signal since there are less cells to produce the luciferase as a response to the 2,4-DNT. Gel thickness and cell concentration should be optimized regarding the optimal sensor output. In summary it is evident that aeration of the cells with air is not sufficient to maintain a maximal bioluminescent signal since the penetration depth of the oxygen is too low.

### 3.4. Liquid-Gas Phase Partition Coefficient of 2,4-DNT

Identification of the gaseous 2,4-DNT concentration was needed to evaluate the biosensor sensitivity. The setup described in Section 3.1 was used to analyze the 2,4-DNT equilibrium vapor concentration. Syringe 1 contained a liquid phase (V_CS_ = 2 mL) with 150 mg 2,4-DNT dissolved in it. The equilibrium liquid-vapor concentration was established over 24 h in a defined gas volume (V_CG_ = 48 mL) in syringe 1. The gas phase was then transferred into syringe 2 to extract 2,4-DNT using the stir bar located in syringe 2 over a period of 24 h. The stir bar was analyzed using GC-MS as stated in material and methods and resulted in a 2,4-DNT gas phase concentration of 0.036 µg mL^−1^.

Using Equation 3 the ratio of the two phase volumes could be calculated (β). Equation 4 was then used to determine the partition coefficient (*K*) for 2,4-DNT between the two phases:

(3) $$\beta =\frac{V_{C_{G}}}{V_{C_{S}}}=\frac{48\;\mathrm{mL}}{2\;\mathrm{mL}}=24$$

(4) $$K=\frac{C_{S}}{C_{G}}\cdot \beta =\frac{150\;\mathrm{mg}\times 50{\;\mathrm{mL}}^{-1}}{0.0018\;\mathrm{mg}\times 50{\;\mathrm{mL}}^{-1}}\times 24=2\times 10^{6}$$

The partition coefficient (*K*) is constant for a compound as long as the temperature and sample matrix are the same. Therefore, the calculated *K* value was used for the estimation of 2,4-DNT headspace concentrations in following experiments.

### 3.5. Basic Biosensor Development

**Figure 5:**
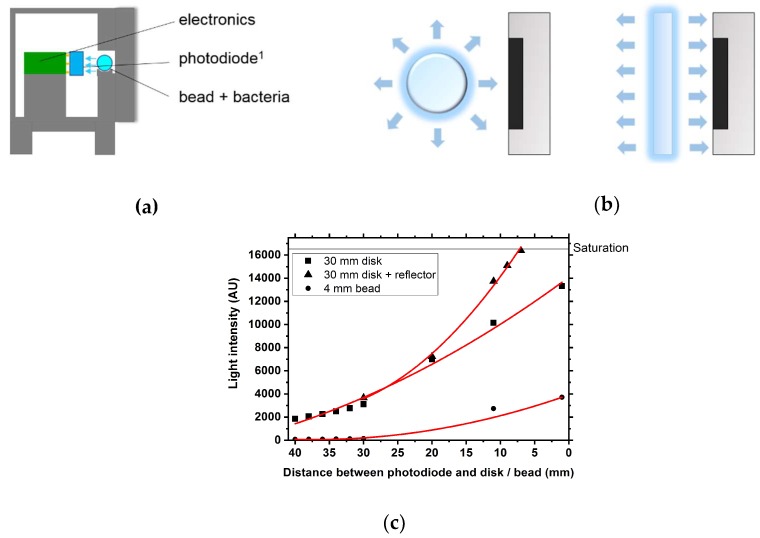
Overview of biosensor prototype 1.0 and its features including an alginate bead containing the bioluminescence bio reporter strain and a photodiode to detect the emitted bioluminescence (**a**). Schematic comparison of the radiation surfaces of a bead and a disk containing the bioluminescent reporter strain (**b**). Light intensity of an illuminated PVDF (Polyvinylidene fluoride) disk (Ø = 30 mm) and an illuminated bead (Ø = 4 mm) at different distance between the object and a photodiode. Additionally, the photodiode was embedded in a reflector and the resulting light intensity was measured and compared to a sole disk (**c**).

In biosensor prototype 1.0 bacterial cells were immobilized in a Ø = 4 mm calcium alginate bead which was placed in front of a photodiode to measure emitted bioluminescence. First experiments were carried out using a pre-induced culture (100 mg∙L^−1^ 2,4-DNT for 3 h) and an agar bead was cast. The bead was placed in biosensor prototype 1.0 but no bioluminescence signal could be measured. The immobilization technique with agar beads did not use the bioluminescent cells effectively. Only a small area of the alginate sphere delivered the emitted light to the photodiode, the rest of the emitted light vanished in the surroundings. A more efficient use of the bioluminescence would be the use of a hydrogel disk (see Figure 5a for comparison). To increase the radiation surface and use it more effectively a new approach using a hydrogel disk was made (biosensor prototype 2.0). As oxygen plays a crucial role in the luciferase reaction bigger beads are not a suitable option, since the oxygen supply in the core of the bead is extremely limited.

This approach enabled the use of optical components like lenses in front of the disk (or mirrors behind) followed by the photodiode due to the planar disk surface. Optical components such as different lens types and reflectors were tested, verifying that reflectors are clearly superior to lenses in terms of output signal. By using a reflector the signal could be increased by up to 40 % compared to the same set-up without a reflector (Figure 5b). Another approach to use the emitted light more efficiently would be to use the other side of the disk as well. At low cell densities (<OD_600_ = 5) light is still able to pass through the hydrogel and could be reflected back towards the photodiode using an additional reflector. With these findings a new biosensor prototype was designed and 3D-printed. The new design featured a gel-cassette to hold the immobilized cells. The cassette was then placed in a closed barrel-like case in a way that the distance between the gel and the photodiode was minimal. One reflector is surrounding the integrated photodiode, the other one is fixed to the lid which closes the case from the cassette entry-side.

### 3.6. Biosensor Measurements

For first proof of principle experiments of the biosensor prototype 2.0, experiments using liquid culture were carried out, using a 3D-printed transparent disk-shaped cuvette (diameter = 30 mm, optical path = 3 mm). Previously induced culture (OD_600_ = 5) was then transferred into the cuvette and placed in the sensor. The entire setup was shaken at 180× rpm and the sensor signal was recorded. as in the fluorescence spectrometer could be observed regarding the initial bioluminescence flash. 

**Figure 6:**
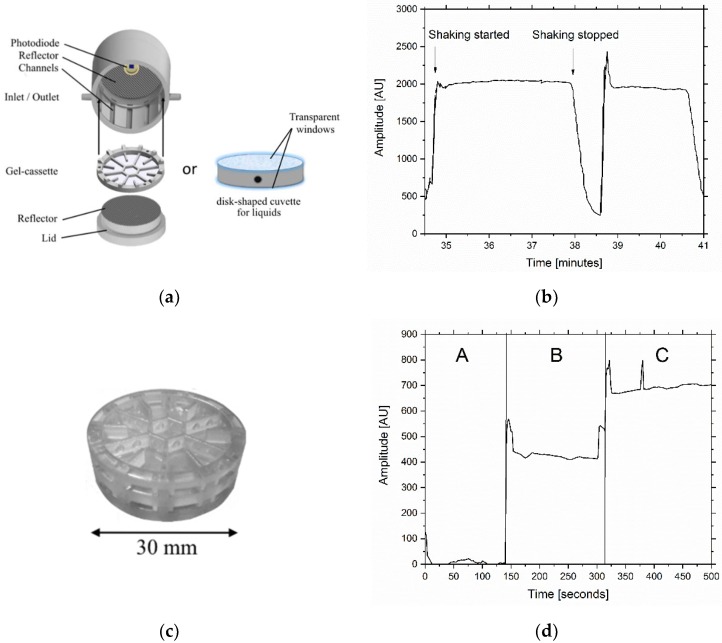
Scheme of prototype 2.0 and its features to measure the bioluminescence of cells in liquid broth and of immobilized cells (**a**). The cells were previously induced with 100 mg∙L^−1^ 2,4-DNT and harvested after 3 h of growth. The liquid culture was transferred into the 3D-printed disk-shaped cuvette and placed in prototype 2.0. The whole sensor was shaken at 180× rpm to ensure oxygen aeration (**b**). Prototype 2.0 was also capable of measuring bioluminescence of immobilized cells using 3D-printed gel-cassettes which could be stacked upon each other to measure two gels in a row (**c**). Therefore, previously induced bacterial cells were immobilized in a 1 % (*w*/*v*) agarose gel disk and placed in the cassette. The bioluminescence was measured using a gel without induced bacterial cells (A), one gel with induced cells (B) and two gels with induced cells (C) (**d**).

The baseline signal was measured at 600 rel. AU. After an initial bioluminescence signal peak of 2500 rel. AU (at 23 min) was observed, the signal returned to a steady baseline at 2000 rel. AU (see Figure 6b). It is notable that the same effect.

For the first time, the weak bioluminescence of the recombinant cells could be recorded using prototype 2.0. As a next step, the cells were immobilized in 1% (*w*/*v*) agarose (final OD_600_ = 5) and the same measurement was repeated. This time the agarose disk containing the immobilized cells was placed in a 3D-printed gel-cassette (Figure 6c). Measurement of one agarose gel disk resulted in a signal up to 500 rel. AU (Figure 6d, B). The signal was further improved by using a second disk behind the first one which resulted in a signal of 700 rel. AU (see Figure 6d, C).

**Figure A8:**
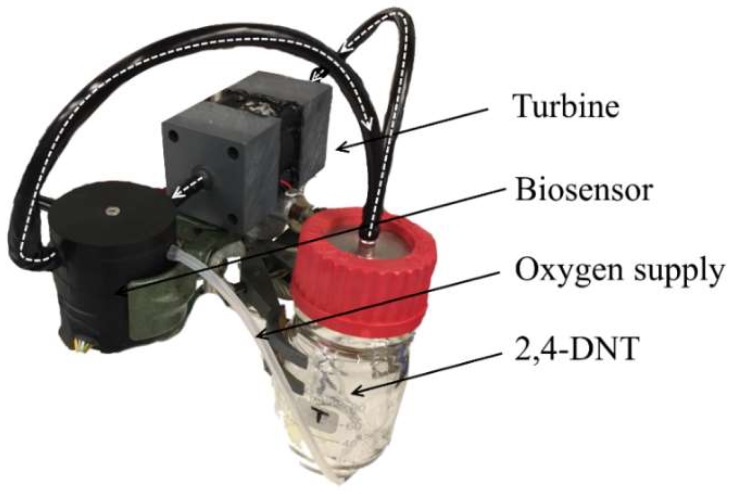
Experimental set-up for the induction of luciferase production with gaseous 2,4-DNT in the biosensor prototype 2.0. The biosensor 2.0 was connected to a turbine which transported 2,4-DNT saturated air from a closed vessel into the sensor and into the vessel to create a closed system (white arrows). The oxygen supply was uncoupled from the 2,4-DNT flow using different gas entries located at the top of the biosensor.

Compared to the measurement in liquid phase 72% of the emitted bioluminescent light is lost if the cells are immobilized in a gel at the same cell density (OD_600_ = 5). Like in the previous experiment gene expression was pre-induced. It was unclear whether production of luciferase could be observed if gaseous 2,4-DNT is introduced into the sensor. Consequently, biosensor prototype 2.0 was modified to enable introduction of both oxygen and 2,4-DNT enriched air to induce luciferase production. A glass vessel was filled with 1 g 2,4-DNT and 100 µL water in order to moisture the 2,4-DNT crystals and enable evaporation. The vessel was then connected to an air pump which directed the air containing 2,4-DNT directly into the sensor structure and onto the agarose disk containing the cells (see Figure A8 for the experimental set-up).

The bioluminescence signal was recorded for 3 h, but no increase in the signal could be observed. It is possible that the non-polar and extremely hydrophobic 2,4-DNT did not diffuse properly into the agarose hydrogel. Moreover, the gel disk showed shrinkage due to dehydration. It is also possible that the cells were unable to respond to the presence of the inducer, since no nutrients were available to maintain metabolism. Consequently, to avoid gel shrinkage and dehydration the gel thickness was increased. Moreover, an increased gel thickness and surface would also increase the bioluminescence signal. Biosensor prototype 3.0 was constructed and 3D-printed using the previously gathered information.

### 3.7. Improved Biosensor System

**Figure 7:**
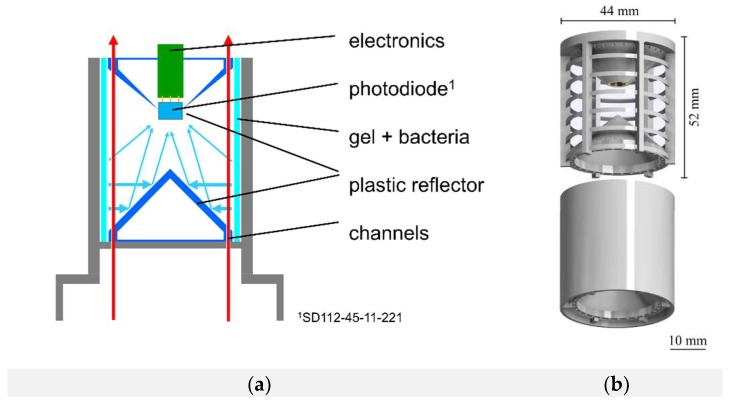
Working principle of the 2,4-DNT biosensor prototype 3.0. The sensor was placed upon contaminated soil and 2,4-DNT rich air is transported into the sensor via a miniature fan. Gene expression is triggered by 2,4-DNT and luciferase is produced. Emitted bioluminescence is focused with the help of two conic reflectors which reflect the light into the embedded photodiode (**a**). Prior to measurements, recombinant cells were immobilized within an agarose hydrogel matrix held by a cage and placed in a case (**b**).

**Figure 8:**
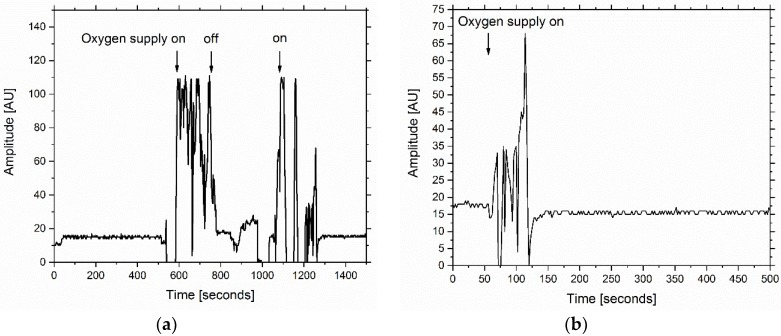
A 1% (*w*/*v*) agarose gel including the bacterial cells was casted with a final cell density of OD_600_ = 5. The whole sensor set-up was then placed in a closed glass vessel on a platform with humidified 2,4-DNT underneath (distance = 5 cm). The vessel was closed and incubated for 3 h. Afterwards molecular oxygen was introduced into the sensor set-up and the resulting bioluminescence response was measured. (**a**) The same experiment was repeated, however no 2,4-DNT was added to the glass vessel. (**b**).

Biosensor prototype 3.0 represented a different set-up than the previous prototypes. In this version the hydrogel was not arranged planar to the photodiode but tubular at a total length of 5 cm. The bioluminescent light was then emitted towards two conic plastic reflectors with a reflection angle of 40° (Figure 7). Thus, emitted light was directly reflected into the photodiode which is embedded in one of the two reflectors. The total surface area of the agarose gel in prototype 3.0 is 31.42 cm^2^ in contrast to a surface area of 9.42 cm^2^ per agarose disk (in the previous prototype). This leads to an enlarged radiation surface (3.3-fold) which also increased the emitted bioluminescence, thus the sensitivity of the biosensor. The gel containing the bacterial cells was cast in a 3D-printed cage which also held the gel. The biosensor was placed in the same glass vessel as used in the previous experiment on a platform 5 cm above 1 g of 2,4-DNT crystals dissolved in 10 mL deionized water. The biosensor was incubated for three hours and the bioluminescence signal was measured afterwards. The use of a stir bar for 2,4-DNT extraction and GC-MS was not advisable in this setup, since the continuous extraction would falsify the actual 2,4-DNT gas phase concentration. Based on the calculated partition coefficient an estimation of the present 2,4-DNT gas phase concentration was established. Figure 8a shows the result of this experiment. After starting the measurement a constant bioluminescence signal at 19 AU was observed, which could be identified as the signal baseline. When turning on molecular oxygen supply an increase of bioluminescent activity up to 110 AU was monitored. The bioluminescence decreased rapidly when oxygen supply was turned off. The initial bioluminescence peak could be reproduced when oxygen was introduced again (see Figure 8a at 1000 s). When turning off oxygen supply the bioluminescence decreased to the base value of about 20 rel. AU. The experiment was repeated as stated above, however no 2,4-DNT was used in the experimental setup (Figure 8b). When oxygen was introduced into the system, a bioluminescence peak was observable (70 AU) which was significantly lower than in the previous set-up. The bioluminescence peak in the negative control could be attributed to operon leakage.

**Figure A9:**
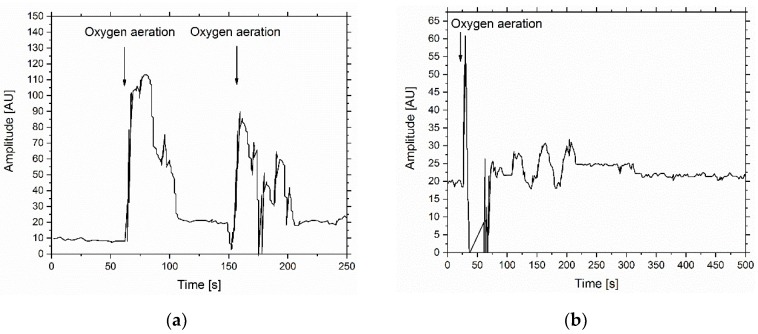

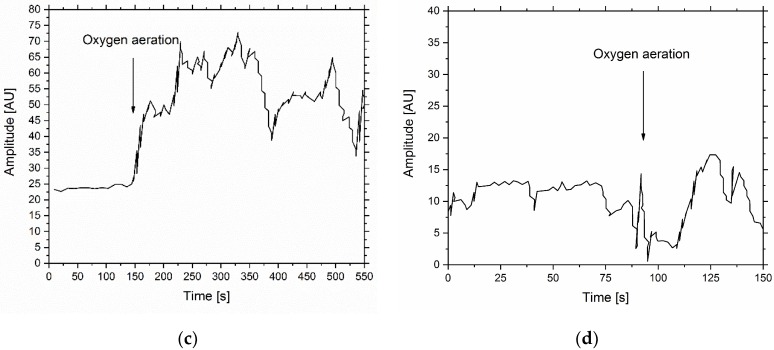
A 1% (*w*/*v*) agarose gel including the bacterial cells was casted with a final cell density of OD_600_ = 5. The whole sensor set-up was then placed in a closed glass vessel on a platform with humidified 2,4-DNT underneath (distance = 5 cm) (**a**,**c**). As a negative control, a 1% (*w*/*v*) agarose gel from the same bacterial culture was casted with a final density of OD_600_ = 5. The gel was added to prototype 3.0 and the whole setup was placed in a closed glass vessel. No 2,4-DNT was added to the bottom of the vessel (**b**,**d**). The vessels were closed and incubated for 3 h. Afterwards molecular oxygen was introduced into the sensor set-up and the resulting bioluminescence response was measured. (**a**) and (**b**) show the first replicate, (**c**) and (**d**) the second replicate.

This experiment was repeated two times with the same parameters as stated. The replicates also indicated that induction of luciferase production was induced with 2,4-DNT and a distinctly higher bioluminescence peak was measurable compared to the negative control (Figure A9). In one replicate (Figure A9c) the bioluminescence peak showed a more stable behavior than in the other replicates.

An estimation of the unknown 2,4-DNT gas phase concentration could be carried out using equation 4 and the net weight of 2,4-DNT in the experimental setup using biosensor prototype 3.0 (C_0_ = 1000 mg 10 mL^−1^). Ratio of the two phase volumes was calculated as stated in Equation (3), however with the volume of the used glass vessel in this particular experiment (V = 100 mL), resulting in a β-value of 999. The concentration 2,4-DNT in the glass vessel where a bioluminescent response was measured was determined to be 49.97 ppb using Equation (5).

(5) $$C_{G}=\frac{C_{0}}{\left(K+\beta \right)}=\frac{1000\;mg\cdot 10\;mL^{-1}}{\left(2\times 10^{6}+999\right)}=4.9975\times 10{\;}^{-5\;}mg\cdot mL^{-1}=49.97\;ppb$$

As hypothesized the initial bioluminescence signal flash could be used as an advantage for detection purposes. As a negative control the same experiment was repeated in a glass vessel without 2,4-DNT. When oxygen was introduced into the sensor, a bioluminescence peak could be observed again. However, the peak was twofold lower than in the experiment with 2,4-DNT and could be explained due to operon leakage. An extremely small amount of luciferase is continuously produced even if no 2,4-DNT is present.

## 4. Discussion

In this study a biosensor for the detection of 2,4-DNT emanating from buried landmines was constructed. The detection of gaseous 2,4-DNT was successful using a recombinant *E. coli* reporter strain, molecularly engineered to sensitively detect this compound [13,14]. Production of luciferase is induced by 2,4-DNT and the emitted bioluminescence is measured by a photodiode, integrated into a specially designed sensor chamber the size of a beverage can. As expected, oxygen had a strong impact on the bioluminescence response. This was verified both in liquid culture and in cells embedded in an agarose hydrogel. It is estimated that up to 20% of the total oxygen consumption of strong luminescent cells may be attributed to the luciferase reaction, indicating the need for a constant supply of oxygen to the system [17]. Immediately following the initiation of aeration, an intermediate strong peak was observed in both liquid culture and in immobilized cells. Bioluminescence flashes in liquid were first observed by Harvey et al. and were extensively examined by different authors [18]. In terms of biosensor construction purposes the flash offers the ability to further increase the sensitivity if needed by introducing oxygen at a short time period which leads to increased bioluminescence emission.

Additionally, it could be shown that temperature has a notable impact on luciferase activity with the maximal bioluminescence at 34 °C. The majority of landmines are buried in countries located around the equator—where temperatures above 30 °C are regular [19]. At these temperatures the dehydration of the gel should be considered. Moreover, higher temperatures pose another problem since the headspace concentration of 2,4-DNT is strongly influenced by the soil moisture content. A high soil moisture content can result in 2,4-DNT headspace concentrations up to five orders the magnitude higher and should be considered [20].

Due to low solubility in water (<0.1 mg mL^−1^) 2,4-DNT was dissolved in 80% acetonitrile for induction. It is reported that organic solvents are able to modulate the bioluminescent response in *luxCDABE* systems by membrane perturbations and release of fatty acids [21]. However, effects of acetonitrile on bioluminescence could be precluded since the organic solvent was strongly diluted and only added during induction. Moreover, in experiments using prototype 3.0 2,4-DNT was humidified in water and no acetonitrile was present. Consequently, the bioluminescence response in these experiments can be attributed to induction effects and not by modulation of acetonitrile.

To increase stability of the cells immobilization in hydrogel matrices is recommended due to a more stable environment and nutrient supply [22]. Moreover, use of hydrogels are feasible since they enable a close proximity of the bioreceptor to the transducer [12,23]. In addition, immobilization exhibits advantages compared to using liquid culture broths. Hydrogels provide structural support and an inert cell surrounding and immobilization enable long term measurements and cell stability. Sensitivity of immobilized cells is nearly the same as in suspension [12]. In previous efforts made to construct a biosensor for 2,4-DNT, the long term stability (temperature wise) and nutrient supply was problematic [24]. Using our hydrogel-approach, these problems are solved. Unfortunately, it is known that oxygen transfer rates in hydrogels are much lower compared to shaken or stirred bioreactors using liquid media [25,26]. Therefore, the oxygen supply in different hydrogel depths was measured. It was shown that the aeration with air is not sufficient to provide adequate oxygen supply even at the outer layers of the gel (<50 µm) at cell densities OD_600_ ≥ 5 which are feasible to increase bioluminescence emission. Aeration with molecular oxygen is needed to guarantee oxygen supply in gel depths up to 400 µm. It is hypothesized that a higher optical density of cells leads to shorter penetration depths of oxygen since more oxygen is consumed already at the outer layers of the gel. On the one hand, a higher cell density is more suitable in terms of higher luciferase concentration for the sensor since it creates a higher output signal. One the other hand this leads to problems regarding oxygen supply in higher gel depths. It is possible that diffusion limitation is present at higher cell densities when the conversion rate of oxygen is higher than the oxygen transfer rate (OTR) especially during growth. Since bacterial cells have a high demand for nutrients and oxygen this might be the case. Therefore, at the tested cell density of OD_600_ = 5 even at 50 µm no oxygen is present when aerating with air. Small solid phase oxygen sources are feasible to maintain the small size of the biosensor and overcome oxygen limitations. Sodium perborate species with Tetraacetylenethylendiamin (TAED) release oxygen and offer a cheap and effective method to maintain metabolic activity and substrate for the luciferase reaction [27]. However, these chemicals are also highly toxic for the cells and should not be in direct contact. If needed and affordable hydrogel-perfluorocarbon composites could also be used to maintain constant oxygen supply in hydrogels [28]. Over different stages of biosensor development it could be shown that the radiation surface of the hydrogel containing the bioluminescent cells is crucial in aspects of sensitivity. An increased surface and volume offers higher bioluminescence signals and a higher long term cell vitality for the incorporated cells.

In the final biosensor prototype the hydrogel was orientated tubular (regarding the photodiode) instead of planar, like in the previous prototype which lead to a 3.3-fold increased radiation surface than in the previous biosensor version. The use of conic reflectors instead of planar ones united the properties of lenses and reflectors since they gather light from a broad acceptance angle and focus it by reflection. It was estimated that 49 ppb of gaseous 2,4-DNT was detected by the constructed biosensor prototype 3.0 using calculations based on the gas-liquid partition coefficient [29]. Since 2,4-DNT is a hydrophobic compound and has a low vapor equilibrium concentration (171.4 ppb) it was expected that a high K-value would be obtained. However, the calculated 2,4-DNT vapor concentration is only an estimation. The headspace concentration of 2,4-DNT above landmines is estimated to be between 20–40 ppt, which would not be detectable using the current biosensor setup [6]. One approach to increase sensitivity could be the adsorption of the nitro aromatic compound to an adsorbent solid phase such as activated coal and the sudden release via UV followed by the induction of the bioreceptor to improve the sensitivity [30]. In next steps different accumulation steps for the volatile 2,4-DNT should be evaluated and integrated into the sensor to further increase the sensitivity. Moreover, optimizations regarding the sensitivity of the biological component are feasible since operon leakage could be observed in the final sensor experiment using a non-induced bacterial culture. The detection of gaseous 2,4-DNT using prototype 3.0 showed reproducibility with restrictions regarding the ground noise and peak behavior. In three replicates the ground noise varied between 10–25 AU. The transimpedance amplifier of the photodiode was set to its maximal value of 8 resulting in a higher signal-to-noise ratio. In future prospects, it is feasible to aim at lower amplifier values to reduce ground noise and guarantee a higher reproducibility in terms of the bioluminescence peak value. Additionally, the selectivity of the bioreporter should be evaluated. It could be shown that induction of the *yqjF* promotor most likely occured via degradation products of 2,4-DNT and not the compound itself. Therefore, the degradation products should be studied intensively to preclude cross-selectivity with other related compounds [14].

As a next step, prototype 3.0 should be tested with 2,4-DNT spiked soil samples to verify whether the constructed sensor will be able to perform in real-world applications. As already mentioned, the soil humidity is a crucial factor in terms of 2,4-DNT evaporation. Consequently, different moisture contents should be evaluated in these preliminary tests. Moreover, weather conditions such as wind velocity and atmospheric conditions play a crucial role in the formation of the boundary layer above the soil where 2,4-DNT is present. All these factors could heavily impact the detection process and should be investigated thoroughly in future experiments. In conclusion, due to the high specify of the inducer binding site of the operon false positive results can be precluded in contrast to other state of art chemical trace sensors. Moreover, the biosensor is easy to handle and can be placed by uninstructed personal. Another advantage of the sensor is its flexibility, as the bioreceptor can be easily exchanged. Any bioreceptor that reacts to analytes through bioluminescence emission can be used. For example, the sensor could be used to analyze complex soil samples without previous purification steps due to the high specifity of the biological component and a natural cellular filter for large particles [31]. Since the luciferase reaction is connected to the respiratory chain of the cells, the sensor could also be used for toxicity measurements and monitor bioluminescence activity of immobilized cells over a long period of time.

## Figures and Tables

**Figure A7:**
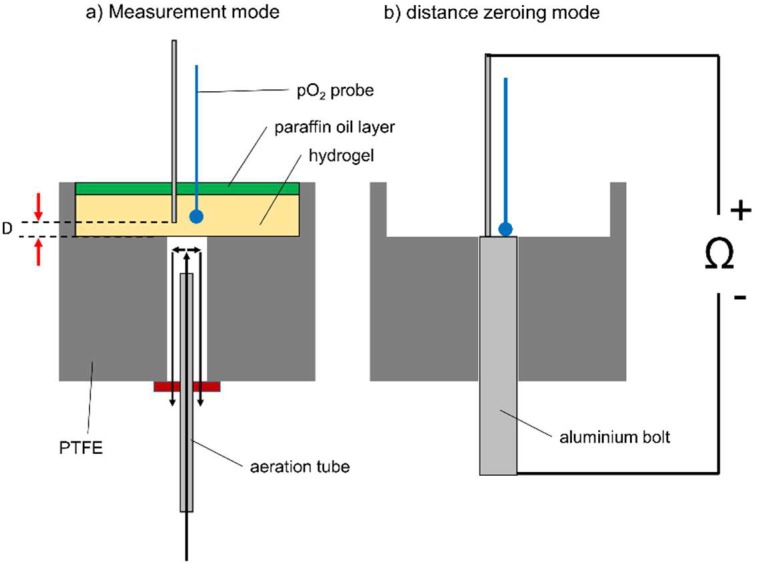
Scheme of the used micromanipulator device to measure oxygen penetration depth in hydrogels. Aeration of the hydrogel was carried out from the bottom of the gel. The gas-filled compartment beneath the gel included two exhaust channels to preclude pressure build-up (**a**). Distance zeroing mode was achieved with an aluminum bolt which was incorporated in the Polytetrafluoroethylene (PTFE) block. A conductive bolt which was fixed to the micromanipulator device was then moved towards the aluminum bolt until a change in resistance was measured. The aluminum bolt was removed and the hydrogel could be casted in the casting mold (**b**).
